# Length of intact plasma membrane determines the diffusion properties of cellular water

**DOI:** 10.1038/srep19051

**Published:** 2016-01-11

**Authors:** Sato Eida, Marc Van Cauteren, Yuka Hotokezaka, Ikuo Katayama, Miho Sasaki, Makoto Obara, Tomoyuki Okuaki, Misa Sumi, Takashi Nakamura

**Affiliations:** 1 Department of Radiology and Cancer Biology, Nagasaki University School of Dentistry, 1-7-1 Sakamoto, Nagasaki 852-8588, Japan; 2 Philips Healthcare, 2-13-37 Kohnan, Minato-ku, Tokyo 108-8507, Japan

## Abstract

Molecular diffusion in a boundary-free medium depends only on the molecular size, the temperature, and medium viscosity. However, the critical determinant of the molecular diffusion property in inhomogeneous biological tissues has not been identified. Here, using an *in vitro* system and a high-resolution MR imaging technique, we show that the length of the intact plasma membrane is a major determinant of water diffusion in a controlled cellular environment and that the cell perimeter length (CPL) is sufficient to estimate the apparent diffusion coefficient (ADC) of water in any cellular environment in our experimental system (ADC = −0.21 × CPL + 1.10). We used this finding to further explain the different diffusion kinetics of cells that are dying via apoptotic or non-apoptotic cell death pathways exhibiting characteristic changes in size, nuclear and cytoplasmic architectures, and membrane integrity. These results suggest that the ADC value can be used as a potential biomarker for cell death.

Diffusion, first observed by IngenHousz in 1789^1^, and later by Brown in 1827^2^, was given a mathematical foundation and a physical interpretation by Einstein in 1905^3^. It is caused by thermal agitation and results in random movement of molecules in a solvent. In a boundary-free medium, the diffusion of molecules depends only on the molecule size, the temperature, and the medium viscosity^4^. In biological tissues, water diffusion is hindered by extracellular and intracellular components, such as fibers and membranes^3^. However, the critical determinants of the diffusion properties in the cellular environment have not been identified.

Several studies have shown that the *in vivo* apparent diffusion coefficient (ADC) is affected by variables such as cellularity, cell size, cell shape, tortuosity, the ratio of extracellular to intracellular water, and the ratio between bound and free water molecules^4^,^5^,^6^,^7^. Recent studies using oscillating gradient (OGSE) diffusion MR imaging provided insight into the heterogeneous structures of biological tissues having different levels of water diffusivity^7^,^8^. However, none of these models could predict the diffusion behavior quantitatively. Therefore, we studied diffusion in a well-controlled cellular environment to identify which property of the cellular environment can independently predict the ADC in our model system.

Cell death can be classified as apoptotic or non-apoptotic on the basis of the morphological appearances, enzymatic criteria, functional properties, and immunological characteristics^9^,^10^. Apoptotic cell death is associated with a rounding of the cell contour, a gradual reduction of cellular volume, chromatin condensation, nuclear fragmentation, and blebbing of the plasma membrane. These morphological changes during apoptotic cell death can cause restricted water diffusivity inside and outside the cells. However, the ultrastructures of the cytoplasmic organelles remain intact, and the cell membrane integrity is preserved until the dying cells are phagocytosed by neighboring macrophages^10^,^11^,^12^.

In contrast, necroptosis, a type of non-apoptotic cell death, is morphologically characterized by increased cellular volume, organelle swelling, and plasma membrane rupture, which is associated with the loss of intracellular content^10^,^11^,^12^,^13^. Cells undergoing necroptosis do not exhibit characteristic chromatin condensation; rather, the chromatin clusters and forms speckles. In addition, necroptosis is marked by early membrane permeabilization and plasma membrane rupture during the later stages. Therefore, we can expect that these morphological changes will increase the water diffusivity inside and outside the cells that are undergoing non-apoptotic (necroptotic) death.

Based on these distinctive morphological characteristics of apoptotic and non-apoptotic cell death, we hypothesized that the molecular diffusion properties of the cells undergoing apoptosis may differ from those dying via the non-apoptotic pathway. Here, we show that the length and integrity of the plasma membrane is a major determinant of molecular diffusion of the cell and that the molecular diffusion kinetics in dying cells differ according to cell death types.

## Results

### Evaluation of the *in vitro* system for measuring diffusion of cellular water in cell pellets

**Figure 1:**
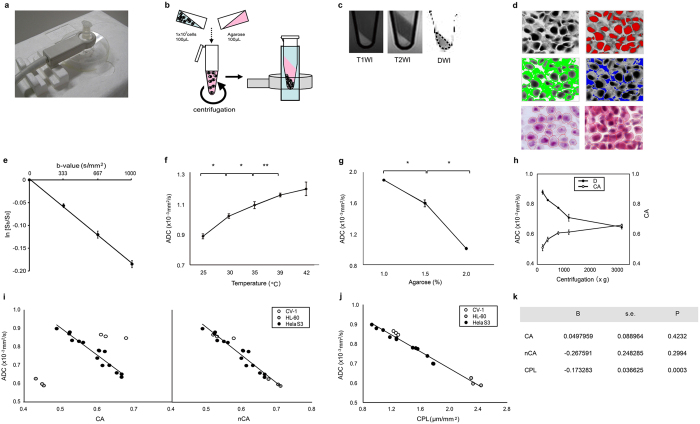
*In vitro* MR imaging to assess the molecular diffusion of the cell. (**a**) Photograph showing an overview of the *in vitro* MR imaging system. (**b**) Schematic drawing showing procedures for preparing a cell pellet from the cell suspension in PBS containing 2% agarose. (**c**) T1- (T1WI) and T2- (T2WI) weighted MR images and molecular diffusion map (ADC map) of a cell pellet at the bottom of an Eppendorf tube. (**d**) Upper and middle panels: Measurement of the cell area (CA) and nuclear-to-cytoplasmic (N/C) ratio. Photomicrographs of a HeLa S3 cell pellet; grey-scale image (upper left panel), nuclear area (red; upper right), cytoplasmic area (green; middle left), and extracellular area (= agarose; blue; middle right). Lower panels: Measurement of the cell perimeter length. Photomicrographs of HeLa S3 pellets. Red closed lines indicate the cell perimeters. (**e**) Graph showing a logarithmic-linear relationship between the b values and the normalized signal intensity. The data are expressed as the mean ± s.e. from 3 independent experiments. (**f**) Graph showing the D values of HeLa cell pellets at varying temperatures. The data are expressed as the mean ± s.d. from 3 independent experiments. *^,^ ** Significantly different (*p < 0.001, **p < 0.05; Tukey-Kramer test). (**g**) Graph showing the ADC values of agarose with varying (0 – 2%) concentrations. Data at each agarose concentration are plotted from 3 independent experiments. * Significantly different (p < 0.001; Tukey-Kramer test). (**h**) Graph showing an inverse relationship between the ADC values (closed circles) and the cell area (CA; open circles) of HeLa S3 cells. The data are expressed as means ± s.d. from 3 independent experiments. (**i**) Graph showing a linear correlation between the ADC values and CA (left panel) or nuclear-weighted CA (nCA, right panel) of HeLa S3 cells. Right panel: nCAs are corrected with the different nuclear-to-cytoplasmic (N/C) ratios of CV-1, HeLa S3, and HL-60 cells. (**j**) Graph showing a linear correlation between the ADC values and the cell perimeter length per pellet area (CPL; μm/μm^2^) of HeLa S3, HL-60, and CV-1 cells. (**k**) Panel showing the statistics of the multiple regression analysis. CA, cell area; nCA, nucleus-weighted percentage cell area; CPL, cell perimeter length; B, partial regression coefficient; s.e., standard error of the mean.

To assess the molecular diffusion of water, we have first established an *in vitro* measurement system for determining diffusion of water in cell pellets (Fig. 1a–d).

A monoexponential relationship between the b values and the signal intensity in logarithmic plots was verified in an experiment with multiple b values (Fig. 1e; Table S1):


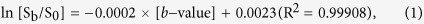


where S_b_ and S_0_ indicate signal intensities at b = 0 s/mm^2^ and b = 333, 667, or 1000 s/mm^2^, respectively. The small standard errors of the mean (s.e.) as evident in the decay curve (Fig. 1e) and the very high R^2^ value indicate that the signal decay can be well-approximated using a monoexponential model, despite the diffusion being hindered^14^. This concept is widely used in clinical imaging and has been proven to be very powerful. The ADC should be distinguished from the free diffusion coefficient D. The close fit also indicates the absence of any perfusion component in the signal.

Accordingly, we performed the measurements using only 2 b factors (0 and 1000 s/mm^2^), which is sufficient for determining the diffusion in our experimental system.

Diffusion is activated at high temperatures and inhibited in high-viscosity solutions. We corroborated these facets in the present model; the ADC values of the cell pellets increased with increasing temperatures and decreased in solutions with increased viscosity (Fig. 1f,g; Tables S2 and S3).

### Intracellular space and diffusion

van Gelderen *et al.*^15^ showed that the ADC of intracellular spaces is approximately 1 order of magnitude lower than that of the extracellular space. Therefore, we corroborated this notion using cell pellets obtained at different centrifugation speeds. Higher centrifugation speeds increased the cell density of the pellet and decreased the ADC values (Fig. 1h; Table S4). We prepared histological sections from the same pellets that were used for determining the percentage cell areas (cell area, CA) of the pellets. Using HeLa S3 cell pellets, we found a linear correlation between the ADC values and the percentage cell area CA (Fig. 1i, left panel; Table S5):


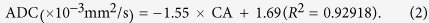


If there is no extracellular matrix (i.e., CA = 1), the ADC value is 0.13 × 10^−3^ mm^2^/s, extrapolating from the abovementioned equation; however, if there are no cells in the tube (i.e., CA = 0), the ADC value is 1.69 × 10^−3^ mm^2^/s. Thus, the equation derived for the relationship between the ADC values and CA is consistent with the results reported by van Gelderen *et al.*

However, the relationships between the ADC and the CA values for other cell lines (CV-1 and HL-60 cells) did not fit the linear correlation observed in HeLa S3 cells (Fig. 1i, left panel; Table S5). The cell lines used in this study have different nucleus-to-cytoplasm (N/C) ratios. The nucleus is considered to have hindered water diffusivity^16^,^17^. Therefore, we tested the notion that differences in N/C ratios affect the molecular diffusion of different cell lines. We determined the mean N/C ratios for the respective cell lines (N/C ratios = 0.388, 0.456, and 0.714 for CV-1, HeLa S3, and HL-60 cells, respectively) and calculated the nucleus-weighted percentage cell areas (nCA; percentage cell area × N/C ratio). We then compared the nCA values with the ADC values. As expected, the nCAs were well-correlated with the ADC values (Fig. 1i, right panel; Table S5):


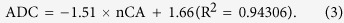


These results indicate that the nuclear size relative to the cell size is another cellular factor that determines diffusion of the cell.

### Cell perimeter length and diffusion

Water diffusion may be severely hindered close to the plasma membrane because of the glycoprotein and glycolipid chains on the outer surface of the plasma membrane and the cytoskeletons on the inner surface of the plasma membrane; these clusters of molecules trap the water molecules of the plasma membrane surface^17^. Thus, it is plausible that longer plasma membranes more severely limit the water diffusivity within biological tissues.

To test the hypothesis that the plasma membrane potentially contributes to large reductions in diffusivity in biological tissues, we measured the cell perimeter length (CPL) of the histological sections obtained from the cell pellets and then compared the CPLs with the ADC values of the same pellets (Fig. 1j; Table S6)


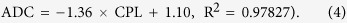


Multiple regression analysis indicated that the CPL alone independently determines the ADC values in all cell pellets (Fig. 1k). Thus, in our model system, CPL is sufficient to calculate ADC.

### Fluorescence correlation spectroscopy (FCS) analysis of living cells in culture

**Figure 2:**
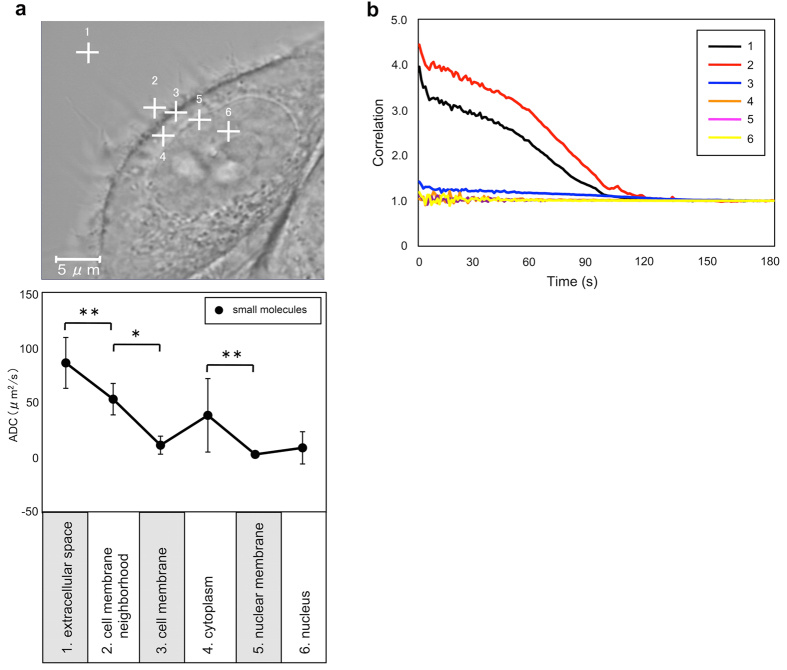
FCS analysis of living cells in culture. (**a**) Upper panel: Confocal microscopy (phase contrast image) showing measurement points in the extracellular (1 and 2) and intracellular (3–6) spaces of HeLa S3 cells in culture. 1, culture medium away from plasma membrane; 2, interphase between the extracellular space and plasma membrane; 3, plasma membrane; 4, cytoplasm; 5, nuclear membrane; 6, nucleoplasm. Lower panel: ADC values of the extracellular and intracellular spaces of living cells in culture. ADC values of the Dio molecules are plotted for 6 measurement points. *^,^ ** Significantly different from the values in the extracellular space (*p < 0.001, **p < 0.05; Tukey-Kramer test). (**b**) Graph showing the FCS autocorrelation curves of Dio molecules in living cells in culture. Each curve was averaged from one location in each of the 6 cellular compartments in a single living cell. Note that, for example, curve 1 (extracellular space) shifts to the right compared with curve 2 (interface between the extracellular space and plasma membrane), indicating that curve 1 has a higher ADC value than curve 2.

To further substantiate the notion that the plasma membrane is a critical determinant for the diffusion properties in a cellular environment, we assessed the diffusivity by using FCS^17^,^18^ (Fig. 2). To monitor the intra- and extracellular water diffusivity in living cells, we traced lipophilic Dio molecules, which were added to the culture medium (phosphate-buffered saline, PBS) and then distributed in the extra- and intracellular spaces of the cell cultures.

FCS revealed that the ADC values of lipophilic Dio molecules in the plasma membrane (11 ± 8 μm^2^/s) were significantly smaller than those of the dye in the culture medium away (≈ 5 μm) from the plasma membrane (86 ± 23 μm^2^/s) (Fig. 2a; Table S7). The ADC values at the interface between the extracellular space and the plasma membrane (53 ± 14 μm^2^/s) were significantly smaller than those of the extracellular space, but these values were significantly greater than those of the plasma membrane. The ADC values of the nucleoplasm (9 ± 15 μm^2^/s) were significantly smaller than those of the cytoplasm (38 ± 33 μm^2^/s), and these values were at levels similar to those of the corresponding membranes. Although careful interpretations are needed because of the large molecular size of rhodamine 6G (C_28_H_31_ClN_2_O_3_ = 479.02) used in the FCS analysis, these results suggest that diffusivity is significantly impeded at the interface between the extracellular space and the plasma membrane as well as within the plasma membrane structure. Sub-micrometer clusters of membrane receptors, which protrude from the membrane surface into the extracellular space in the range of 400-800 nm, may explain the observed slow diffusion area at the interface between the extracelular space and the plasma membrane^19^.

### Diffusion in apoptosis

**Figure 3:**
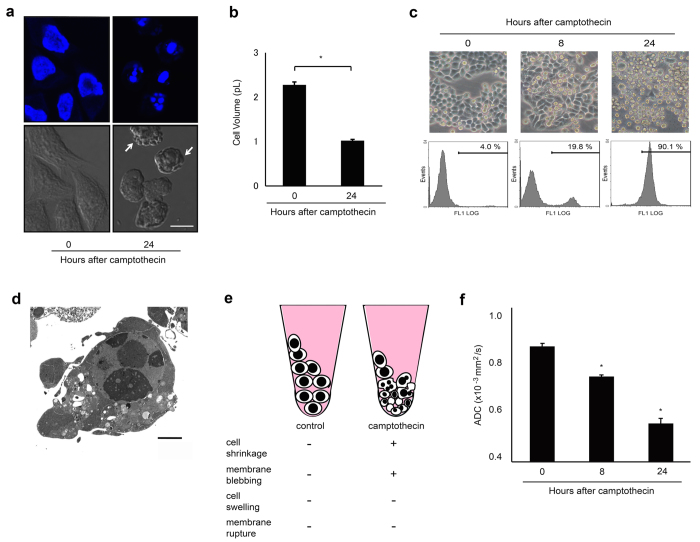
Molecular diffusion of camptothecin-treated (apoptotic) HeLa S3 cells. (**a**) Confocal microscopy for DAPI staining (upper panels) showing the fragmented nuclei of camptothecin-treated (24 h) HeLa S3 cells. Differential interference microscopy (lower panels) showing blebbing (arrows). Scale bar = 10 μm. (**b**) Graph showing decreases in cell volume of camptothecin-treated HeLa S3 cells. * Significantly different from values at 0 h (p < 0.001; t-test). (**c**) Phase-contrast micrographs (upper panels) show non-treated (0 h) or 1 μM camptothecin-treated (8 h and 24 h) HeLa S3 cells. Cells are rounded and detach from the dish bottoms as early as 8 h after the addition of camptothecin in culture medium. FACS analysis (lower panels) show time-dependent increases in annexin-positive cell fraction of camptothecin-treated cells. Bars with percentages in graphs indicate annexin-positive (apoptotic) cell fractions. (**d**) Transmission electron microscopy showing chromatin condensation and blebbing of the intact plasma membrane of camptothecin-treated (24 h) HeLa S3 cell. Scale bar = 2 μm. (**e**) Schematic representation and cellular characteristics of cell pellets containing camptothecin-treated (24 h, apoptotic) HeLa cells. (**f**) Graph showing the time-dependent decreases in ADC values of camptothecin-treated (8 h and 24 h) HeLa S3 cells. All cells that adhered to or detached from the bottom of culture dishes were collected, resuspended in PBS, centrifuged, and assessed for ADC values. The data are expressed as the mean ± s.d. from 3 independent experiments. * Significantly different from the ADC values at 0 h (p < 0.001; Tukey-Kramer test).

Given that the length of the plasma membranes determines the water diffusivity, we next assessed this conjecture by studying the kinetics of the D values during different types of cell death because these processes are well-known and the deformation of the cell membranes during the processes are well-documented. Apoptosis was induced by culturing HeLa cells in the presence of camptothecin or staurosporine^20^. Apoptotic cells underwent cell shrinkage, membrane blebbing, chromatin condensation, and nuclear fragmentation along with cell apoptosis (Fig. 3a–e; Table S8). However, their plasma membranes remained intact even during the final stages of the apoptotic processes, resulting in an increased CPL. The apoptotic cells exhibited a gradual decline in ADC values, compliant with our model (Fig. 3f; Table S9). Similar changes in the kinetics of the ADC values were observed in the staurosporine-treated HeLa cells (Fig. S1; Tables S10-12).

### Diffusion in necroptosis

**Figure 4:**
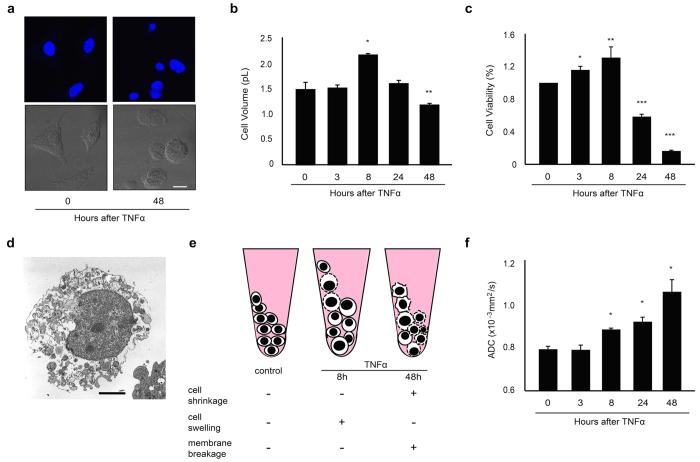
Molecular diffusion of TNFα-treated (necroptotic) L929 cells. (**a**) Confocal microscopy for DAPI staining (upper panels) and differential interference microscopy (lower panels) showing intact nuclei of TNFα-treated (48 h) L929 cells. Scale bar = 10 μm. (**b**) Graph showing early increases (8 h) and subsequent decreases (48 h) in the cell volume of TNFα-treated L929 cells. *^,^ ** Significantly different from the cell volumes at 0 h (*p < 0.001, **p < 0.01; Tukey-Kramer test). (**c**) Graph showing early increases (3 h and 8 h) and subsequent decreases (24 h and 48 h) in the cell viability of TNFα-treated L929 cells. *^,^ **^,^ *** Significantly different from cell viability at 0 h (* p < 0.05, **p < 0.01, ***p < 0.001; Tukey-Kramer test). (**d**) Transmission electron microscopy showing disruption of the plasma membrane and swelling of the mitochondria of TNFα-treated (48 h) L929 cells. Scale bar = 2 μm. (**e**) Schematic representation and cellular characteristics of cell pellets containing TNFα-treated (necroptotic, 8 h and 48 h) L929 cells. (**f**) Graph showing a gradual increase in the ADC value of necroptotic L929 cells.* Significantly different from the ADC values at 0 h (p < 0.001; Tukey-Kramer test).

Necroptosis can be induced in L929 cells by treatment with tumor necrosis factor α (TNFα) or with the pan-caspase inhibitor (zVAD-fmk)^21^,^22^. Necroptosis caused temporary cell swelling at the early stages and cell shrinkage at the later stages after treatment (Fig. 4a–c,e; Tables S13 and S14). In contrast to the apoptotic cells, nuclear fragmentation was not evident in these cells (Fig. 4a). Instead, the mitochondria and endoplasmic reticulum (ER) swelled, and the plasma membranes ruptured (Fig. 4d)^23^. The ADC values of necrotic cells continued to increase throughout the treatment period (Fig. 4f; Fig. S2; Tables S15-18).

### Diffusion in irradiation-induced non-apoptotic cell death

Irradiated U937 cells exhibited mixed features of apoptotic and necrotic cell death (Fig. S3a–d; Tables S19 and S20)^24^. The ADC values of the irradiated U937 cells displayed biphasic changes with decreases during the early stages and increases during the later stages after irradiation (Fig. S3d,f; Table S21).

## Discussion

The exact mechanisms determining the water diffusivity in biological systems remain to be fully elucidated. In the present study, we demonstrated that the plasma membrane is a major determinant of diffusion of water in a cellular system by using an *in vitro* cellular environment measured in a clinical MR imaging system. This notion was supported by the results obtained from the FCS analysis of cell cultures showing that the ADC values near the plasma membrane were smaller than those in the culture medium and that the ADC values near the nuclear membranes were smaller than those in the nucleoplasm. In addition the FCS analysis revealed that the ADC values were significantly decreased at the interface between the extracellular space and the plasma membrane, suggesting the presence of a biolayer with slow diffusivity along the plasma membrane.

Vereb *et al.* proposed an updated Singer-Nicolson model that views the plasma membrane as a dynamic mosaic structure comprising lipid and/or protein clumps^19^. These clumps can rearrange dynamically in response to biological requirements for the cell^25^. In this model, the lateral movement of water (and protein) molecules along the plasma membrane surface is greatly affected by the lipid and protein anchors fixed to the plasma membrane. Many lipids and proteins have short carbohydrate chains that protrude from the plasma membrane surface to form glycoproteins and glycolipids. These chains form hydrogen bonds with the water molecules that surround the plasma membrane surface, and many of them serve as receptor molecules by binding with hormones and neurotransmitters and conveying extracellular signals into the cell^25^. On the inner surface of the plasma membrane, the cytoskeleton and proteins with long intracellular domains are also important in restricting the lateral movement of the water molecules^19^. Therefore, this model implies that large reductions in water diffusion may result from interactions between water molecules and carbohydrate chains and cytoskeletons that protrude into the extracellular and intracellular spaces^25^.

Many factors contribute to water diffusion in biological tissues, such as fibers, membranes, macromolecular complexes and other intracellular structures. In particular, osmotic changes in the cellular environment associated with cell size changes can greatly affect the water diffusivity in the tissues. Hypotonic tissue conditions lead to cell swelling and decreases in tissue ADCs, consistent with the present results obtained using cell pellets^26^,^27^. However, intracellular ADCs of swollen cells in hypotonic conditions are increased probably due to the decreased density of intracellular component and decreased intracellular viscosity^26^,^28^. Therefore, the possible decreases in extracellular ADC caused by increased tortuosity in the extracellular diffusion may surpass the changes in the intracellular ADCs in tissue levels^29^. However, in the present MR imaging system we cannot determine the intracellular and extracellular ADCs separately.

Many researchers have preferred to explain the reduction of water diffusivity in biological tissues based upon the ‘mechanistic theory,’ where the hindered water diffusivity is caused by collisions of water molecules onto the plasma membrane barrier. This notion helps to explain the changes in water diffusivity in the cell pellets with different cell densities and/or cell morphologies in the present study. However, evidence that the plasma membrane impedes the diffusivity by directly blocking the free movement of water molecules has not been established. Furthermore, water diffusion is considered to be allowed in the plasma membrane^25^. Therefore, other factors are important in the current context.

Recent studies have suggested that the plasma membrane permeability greatly affects water diffusivity in cellular structures. Accordingly, water transport through aquaporins (AQPs) can enhance cell swelling, thereby influencing the intracellular water diffusivity without significantly affecting the extracellular tortuosity^30^,^31^. However, the relevance of membrane permeability to water diffusivity in pathology such as apoptosis and necrosis is not clear.

In contrast to the ‘mechanistic theory’ for the reduced water diffusivity in cellular tissues, Le Bihan proposed a new concept that a slow diffusion layer surrounding the charged plasma membrane might hinder water diffusion^32^. We found this concept very useful for understanding the factor(s) that critically contributes to cellular water diffusion and we believe that our findings support this idea. At present, it is not well-understood how the slow diffusion layer is constructed in the close vicinity of the plasma membrane. However, some amino acids, such as arginine (Arg) and lysine (Lys), are negatively charged, and some types of membrane phospholipids, such as phosphatidylserine (PS) are also negatively charged^33^,^34^. Therefore, the plasma membrane can be negatively charged, and increased concentrations of these proteins and lipids can enhance the interaction of ligand and glycoprotein receptors on the plasma membrane surfaces, creating a slow diffusion layer on the membrane surface.

The establishment of an effective detection technique for apoptotic and non-apoptotic cell death is important for the evaluation of the treatment efficacy in patients with cancer. Discriminating necrotic from apoptotic cell death is also important because necrotic cell death is often associated with unwarranted cell loss and can lead to local inflammation via the liberation of factors from dead cells that stimulate the innate immune system^23^. Several biochemical features of apoptosis have been used for the *in vitro* or *ex vivo* detection of the process, including the TUNEL, DNA-laddering, annexin-V, and caspase-activity assays^24^. The *in vitro* detection of non-apoptotic cell death has largely relied on the morphological features. However, the cellular release of 2 biomarkers, chromatin protein high-mobility group B1 and cyclophilin A, specific to necrosis has been reported^20^,^35^. Imaging techniques for differentiating between apoptotic and non-apoptotic cell death are limited^24^. In the present study, we presented the use of diffusion-weighted imaging as a tool for the *in vivo* detection and differentiation of apoptotic and non-apoptotic cell death events. Therefore, diffusion-weighted MR imaging is a powerful tool for diagnosing and managing patients in neurology and oncology and is particularly useful in *in vitro* experiments aimed at the discovery of new drugs for chemotherapy^36^,^37^. However, apoptosis and necrosis may coincide in the same biological tissues. In addition, autophagy, another type of cell death mechanism, can be triggered by common upstream signals^38^,^39^. Therefore, the precise discrimination between different cell death processes may be challenging in clinics.

One of the limitations of this study is that we merely showed correlations between the diffusion results and the cell death processes. However, the present results support the hypothesis that the plasma membrane with protruding glycoproteins that contain negatively charged amino acids can contribute to the decreased diffusivity around the cell. The slow trafficking along the plasma membrane would be beneficial for efficient communications between the extracellular space and the cell This concept is highly speculative at this stage and further experiments are required to establish the charged plasma membrane as the critical factor for cellular diffusion, e.g., using a wide variety of cell lines. In addition, the present *in vitro* system using cell pellets does not allow for assessment of intracellular and extracellular diffusion separately. This shortcoming could be overcome by using an NMR system with much higher magnetic field intensity, increasing the sensitivity to cellular changes in apoptotic vs. non-apoptotic cell death. In this context, we used only 3 b-values, thereby limiting the detailed assessment of cellular water diffusion in a larger attenuation range. Therefore, it would be interesting to probe the multiexponential behavior of intracellular and extracellular water using high (>1000 s/mm^2^) b-values.

In conclusion, we have shown that the length of intact plasma membrane allows for prediction of the ADC of water in a cellular system, regardless of cell types, and that the diffusion kinetics in dying cells differ according to cell death types.

## Methods

### Cell lines and culture

The cell lines used in this study were HeLa S3 (human cervical cancer), HeLa (human cervical cancer), HL-60 (human promyelocytic leukemia), CV-1 (monkey kidney fibroblasts), U937 (human monocytic leukemia), and L929 (mouse fibroblasts). Cells were maintained growing exponentially in DMEM medium supplemented with 10% fetal bovine serum (FBS) (HeLa S3, HeLa, CV-1, and L929) or RPMI1640 medium supplemented with 10% FBS (HL-60 and U937) before use in the following experiments.

### *In vitro* diffusion-weighted MR imaging

MR imaging was performed using a 1.5 T MR system (1.5T Intera Master, Philips Healthcare) equipped with a 23-mm microscopy coil (Philips Healthcare) (Fig. 1a). T1-weighted imaging (TR/TE/number of signal acquisitions = 500 ms/15 ms/2) was performed using a 50-mm field-of-view (FOV), a 1.5-mm slice thickness, and a 224 × 156 matrix size. T2-weighted imaging (TR/TE/number of signal acquisitions = 2000 ms/90 ms/4) was performed using a 50-mm FOV, a 1.5-mm slice thickness, and a 224 × 152 matrix size. Diffusion-weighted MR imaging (TR/TE/number of signal acquisition = 355 ms/96 ms/16) was performed with pulsed gradient diffusion sensitized single-shot spin echo-echo-planar imaging using a 50-mm FOV, a 3-mm slice thickness, and a 128 × 100 matrix size. The b-values varied between 0 and 1000 s/mm^2^. A TE of 96 ms was used for all experiments, Δ was 48.4 ms and δ was 19.9 ms, resulting in a diffusion time (defined as Δ − δ/3) of 41.8 ms. A relatively long diffusion time was chosen so that we were in the hindered diffusion regime for water molecules in the experimental model system we used. Indeed, an order-of-magnitude calculation based on Einstein’s expression for the root-mean-square displacement shows the water molecules will diffuse over a distance in the order of tens of mm. Given this displacement the water molecules are expected to interact with membranes, macromolecules etc. in a cellular environment and their diffusion will be influenced by this interaction. This leads to the diffusion being hindered. This is the base for using diffusion of water to probe the cellular environment, as the change of the apparent diffusion coefficient will be proportional to the number of interaction during the diffusion time. When using this type of MR imaging sequence, the influence of the read-out gradients and other gradients used for localizing on the diffusion sensitizing is small compared to the influence of the diffusion gradients proper and can be ignored^40^.

Cells (1 × 10^7^) were washed and resuspended in 100 μL PBS, transferred into an Eppendorf tube (1.5-mL DNA LoBind Tube) containing 0–2% agarose in 100 μL PBS, and then centrifuged at varying speeds (200–3200 × *g*) (Fig. 1b). The Eppendorf tube was inserted into a broader test tube (12-mm diameter, Beckman Coulter) that was filled with PBS, and the test tube was positioned such that the obtained cell pellet was at the center of the coil (Fig. 1a,b).

An irregular region of interest (ROI) for determining the ADC was manually placed on the obtained diffusion-weighted image so that it included the maximum pellet area, excluding the tube wall (Fig. 1c). T1- and T2-weighted MR images were used as references for determining pellet area.

Perfusion-related incoherent microcirculation can be ignored in the *in vitro* system used in the present study. We corroborated that for the imaging sequence used the relationship between signal intensities and b-values can be expressed using the following equation:


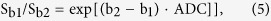


where S_b1_ and S_b2_ are the signal intensities at 2 different b-values. Accordingly, using logarithmic plots, ADC values can be obtained with a linear regression algorithm using b-values of 0 and 1000 s/mm^2^.

### Histomorphometric analysis

Cell pellets were embedded in OCT compound (Tissue-Tek) and frozen in liquid nitrogen. Frozen sections (5 μm thick) were stained with hematoxylin and eosin.

Histomorphometry of cell pellets was performed under a microscope (Axioscope 2 plus, Zeiss) using AxioVision software (AutoMeasure, ver. 4.5, Zeiss). A square observation field (6400 μm^2^) was manually placed on a cell pellet area under a microscope at a magnification of 40×. The CPL is expressed as the total length of the perimeter (μm) per observation field area (μm^2^) (Fig. 1d). The percentage cell area was expressed as the ratio of the total area (μm^2^) of the cell and the total area (μm^2^) of the observation field (Fig. 1e). The nuclear-to-cytoplasmic (N/C) ratio was also calculated by the following formula: [total area of nuclei]/[total area of the cell].

Histomorphometric data obtained from 3 observation fields were averaged for each cell pellet.

### Fluorescence correlation spectroscopy (FCS)

The FCS technique can determine the real-time diffusion kinetics of living cells in culture by analyzing a fluctuating fluorescence signal over time in one location inside the scanning confocal detection volume using temporal correlation algorithm. HeLa S3 cells were seeded onto an 8-well chambered coverglass (Lab-Tek II, Nunc) at a density of 7 × 10^4^ cells/mL and the cells were left to grow overnight in the culture medium (DMEM medium supplemented with 10% FBS). For the FCS study, the culture medium was replaced with PBS, lipophilic Dio (Invitrogen) was added to the PBS (final concentration at 100 nM), and the cultivation was continued at 37 °C throughout the measurement. Confocal images of the cells were collected on an LSM 780 microscope (Zeiss) equipped with a C-Apochromat 40×/1.2 W Corr objective. Excitation was from a 488 nm laser. The pinhole size of the FCS detector was 35 μm. Three sequential readings of 10 s each were performed per measurement point at the extracellular (>5 μm away from the plasma membrane and at the interface between the extracellular space and the plasma membrane) or intracellular (the plasma membrane, cytoplasm, nuclear membrane, and nucleoplasm) space of a cell (Fig. 3a,b). We collected FCS data from 3 cells. The obtained FCS data were processed and analyzed using a Zenn software (Zeiss). The diffusion coefficients of Dio dye were determined by using the following formula:


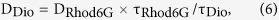


where D_Dio_ and τ_Dio_ are the diffusion coefficient and diffusion time of the Dio dye molecule, respectively, and D_Rhod6G_ and τ_Rhod6G_ are the diffusion coefficient and diffusion time of the rhodamine 6G molecule, respectively.

D_Rhod6G_ is a known parameter (=2.8 × 10^−10^ m^2^/s) and τ_Rhod6G_ was estimated in a 100 nM PBS solution as 30.593 μs under the present FCS set up.

### Induction of cell death

HeLa and HeLa S3 cells were induced to die via apoptotic processes by treatment with staurosporine (1 μM, Sigma) and camptothecin (1 μM, Sigma), respectively. L929 cells were induced to die via necroptosis by treatment with TNFα (20 ng/mL, Sigma) or the pan-caspase inhibitor, zVAD-fmk (N-benzyloxycarbonyl-Val-Ala-Asp-fluoromethylketone) (20 μM, BD Pharmingen). Cell death was also induced by X-irradiation (5-60 Gy, 150 kV, 15 mA, 0.5-mm Al plus 0.2-mm Cu filters) of U937 and HeLa cells.

The extent of cell death was monitored by using the following methods: Apoptosis was assessed after incubating cells with annexin V-FITC (Sigma) at room temperature for 10 min. Cells that were positive for annexin were analyzed using an FACS system (EPICS ADC, Beckman Coulter). For FACS analysis, cells (5 × 10^5^/mL) were collected, washed with PBS, and stained with annexin. Cell viability was assessed by a modified MTT dye reduction assay using WST-8 (2-(2-methoxy-4-nitrophenyl)-3-(4-nitrophenyl)-5-(2,4-disulfophenyl)-2H-tetrazolium, monosodium salt) (Dojindo Molecular Technologies). Cell viability was analyzed using a multi-label counter (Wallac 1420 ARVOsx, PerkinElmer) and is expressed as the ratio of WST-8 values obtained from the treated cells relative to untreated cells.

### DAPI staining

Changes in the nuclear morphology of dying cells were assessed by DAPI staining. Cells were washed with PBS and fixed with 4% paraformaldehyde in PBS for 20 min and were then permeabilized with 0.2% Triton X-100 in PBS for 15 min at room temperature. Visualization of the nuclei was achieved by incubating the cells with DAPI (1 μg/mL) for 10 min at room temperature. Immunofluorescent visualization was performed under a TCS SP2 AOBS confocal microscope (Leica Microsystems).

### Transmission electron microscopy

Apoptotic and non-apoptotic cell death were also morphologically determined using transmission electron microscopy (TEM). For the TEM assessment of dying cells, cell pellets (1 × 10^7^ cells) were fixed with 2% glutaraldehyde in 0.1 M PBS solution at 4 °C, stained with 2% OsO_4_, and embedded in Epon 812 resin. Ultrathin sections (80 nm) were observed under a JEM-2000EX transmission electron microscope (JEOL).

### Measurement of cell size

The average diameters (μm) and volumes (pL) of the cells were determined using an automated cell counter (Scepter 1.2; Millipore).

### Statistical analysis

The Mann-Whitney U test or Tukey-Kramer test was used for comparing the D values, cell volumes, and cell viability between the different cultures. Multiple regression analysis was performed to assess the correlation between the D values and the cellular parameters. The statistical analyses were performed using SPSS (Version 18.0, IBM Corporation).

## Additional Information

**How to cite this article**: Eida, S. *et al.* Length of intact plasma membrane determines the diffusion properties of cellular water. *Sci. Rep.* **6**, 19051; doi: 10.1038/srep19051 (2016).

## Supplementary Material

Supplementary Information
