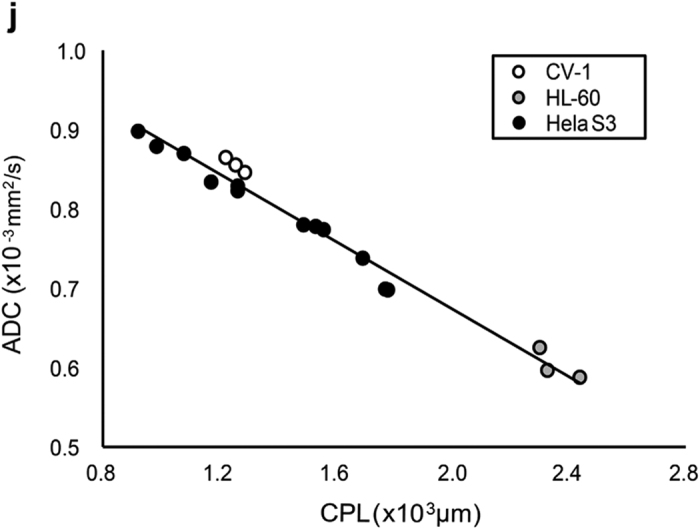# Corrigendum: Length of intact plasma membrane determines the diffusion properties of cellular water

**DOI:** 10.1038/srep25681

**Published:** 2016-05-20

**Authors:** Sato Eida, Marc Van Cauteren, Yuka Hotokezaka, Ikuo Katayama, Miho Sasaki, Makoto Obara, Tomoyuki Okuaki, Misa Sumi, Takashi Nakamura

Scientific Reports 6: Article number: 19051; 10.1038/srep19051 published online: 01112016; updated: 05202016.

This Article contains errors.

In Figure 1j, the x-axis ‘CPL(×10^3^ μm)’ was incorrectly given as ‘CPL(μm/mm^2^). The corresponding Figure legend,

“(**j**) Graph showing a linear correlation between the ADC values and the cell perimeter length per pellet area (CPL; μm/μm^2^) of HeLa S3, HL-60, and CV-1 cells.”

should read:

“(**j**) Graph showing a linear correlation between the ADC values and the cell perimeter length per pellet area (CPL; μm) of HeLa S3, HL-60, and CV-1 cells.”

The correct Figure 1j appears below as Figure 1.

In the Results section under subheading ‘Cell perimeter length and diffusion’ Equation (4),
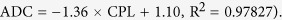


should read:
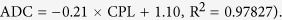


## Figures and Tables

**Figure 1:**