# Supplementary material for: Length of intact plasma membrane determines the diffusion properties of cellular water
Source: Sci Rep. 2016 Jan 11;6:19051. doi: 10.1038/srep19051 (PMC4707473; doi:10.1038/srep19051)
Supplement: Supplementary Information [file srep19051-s1.pdf]

Length of intact plasma membrane determines the diffusion properties of cellular water

Sato Eida[1,3], Marc Van Cauteren[2,3], Yuka Hotokezaka[1,3], Ikuo Katayama[1], Miho Sasaki[1], Makoto Obara[2], Tomoyuki Okuaki[2], Misa Sumi[1], Takashi Nakamura[1,4]

1. Department of Radiology and Cancer Biology, Nagasaki University School of Dentistry,1-7-1 Sakamoto, Nagasaki 852-8588, Japan

2. Philips Healthcare, 2-13-37 Kohnan, Minato-ku, Tokyo 108-8507, Japan

3. These authors contributed equally to this work.

4. Corresponding author

Table S1 (Figure 1e) Logarithmic-linear relationship between the b values and the normalized signal intensity				
	b-value (s/mm^2)			
	0	333	667	1000
ln[S_b/S_0] for				
experiment 1	0	-0.052	-0.113	-0.176
experiment 2	0	-0.058	-0.123	-0.188
experiment 3	0	-0.060	-0.126	-0.191
mean	0	-0.057	-0.120	-0.185
s.e.	0	0.004	0.008	0.008

Table S2 (Fig. 1f) D values of HeLa cell pellets at varying temperatures					
	temperature (°C)				
ADC (x 10^{-3} mm^2/s)	25	30	37	39	42
	0.89	1.00	1.07	1.16	1.14
	0.90	1.03	1.11	1.14	1.23
	0.87	1.02	1.08	1.16	1.21
mean	0.89	1.02	1.09	1.15	1.19
s.e.	0.02	0.01	0.02	0.01	0.05

Table S3 (Fig. 1g) ADC values of agarose with varying (0 – 2%) concentrations			
	agarose (%)		
ADC (x 10^{-3} mm^2/s)	1.0	1.5	2.0
	1.90	1.60	1.02
	1.90	1.64	1.02
	1.90	1.55	0.99
mean	1.90	1.60	1.01
s.e.	0.00	0.05	0.02

Table S4 (Fig. 1h) Relationship between ADC values and cell area of HeLa S3 cells					
	centrifugation (×*g*)				
	200	400	800	1200	3200
ADC (x 10^{-3} mm^2/s)					
	0.90	0.83	0.78	0.74	0.66
	0.88	0.83	0.78	0.70	0.65
	0.87	0.82	0.77	0.70	0.64
mean	0.88	0.83	0.78	0.71	0.65
s.e.	0.01	0.01	0.00	0.02	0.01
CA					
	0.49	0.53	0.60	0.60	0.65
	0.52	0.55	0.60	0.63	0.67
	0.52	0.56	0.62	0.61	0.67
mean	0.51	0.55	0.61	0.61	0.66
s.e.	0.02	0.02	0.01	0.02	0.01

**Table S5 (Fig. 1i) Correlation between ADC values and cell area (CA) or nuclear-weighted CA of HeLa S3 cells**			
cell line	CA	nCA	ADC (x 10^{-3} mm^2/s)
CV-1	0.61	0.53	0.86
	0.68	0.58	0.85
	0.62	0.52	0.86
HL-60	0.43	0.68	0.63
	0.45	0.70	0.60
	0.45	0.71	0.59
HeLa S3	0.49	0.49	0.90
	0.52	0.52	0.88
	0.52	0.52	0.87
	0.53	0.53	0.83
	0.55	0.55	0.83
	0.56	0.56	0.82
	0.60	0.60	0.78
	0.60	0.60	0.78
	0.62	0.62	0.77
	0.60	0.60	0.74
	0.63	0.63	0.70
	0.61	0.61	0.70
	0.65	0.65	0.66
	0.67	0.67	0.65
	0.67	0.67	0.64

Table S6 (Fig. 1j) Correlation between ADC values and cell perimeter length

cell line	perimeter (μm)	ADC (x 10^{-3} mm^2/s)
CV-1	1287	0.85
	1255	0.86
	1221	0.85
HL-60	2325	0.60
	2436	0.59
	2299	0.63
HeLa S3	984	0.88
	921	0.90
	1078	0.87
	1263	0.82
	1263	0.83
	1171	0.83
	1530	0.78
	1557	0.77
	1488	0.78
	1692	0.74
	1769	0.70
	1778	0.70

Table S7 (Fig. 2a) Fluorescence correlation spectroscopy analysis of Dio molecules in living cells in culture

	subcompartment					
	1	2	3	4	5	6
ADC ($\mu m^2/s$)						
	74.6	52.9	14.6	74.3	1.0	0.7
	112.6	37.6	17.5	19.5	2.7	0.4
	71.0	40.9	2.1	2.2	2.5	25.9
		63.2		57.9	6.8	
		71.1				
mean	86.0	53.1	11.4	38.5	3.2	9.0
s.e.	23.1	14.3	8.2	33.3	2.5	14.6

**Table S8 (Fig. 3b) Decreases in cell volume of camptothecin-treated HeLa S3 cells**		
	cell volume (pL)	
	control	24 h
	2.2	1.0
	2.2	1.0
	2.3	1.0
mean	2.2	1.0
s.e.	0.1	0.0

**Table S9 (Fig. 3f) Time-dependent decreases in ADC values of camptothecin-treated (8 h and 24 h) HeLa S3 cells**			
	ADC (x 10^{-3} mm^2/s)		
	control	8 h	24 h
	0.90	0.76	0.57
	0.87	0.75	0.52
	0.88	0.75	0.56
mean	0.88	0.75	0.56
s.e.	0.01	0.01	0.02

**Table S10 (Fig. S1b) Decreases in cell volume of staurosporine-treated HeLa cells**		
	cell volume (pL)	
	control	24 h
	3.10	1.69
	3.33	1.83
	3.23	1.55
mean	3.22	1.69
s.e.	0.16	0.14

**Table S11 (Fig. S1c) Decreases in cell viability of staurosporine-treated HeLa cells**		
	cell viability (vs. control)	
	control	24 h
	1	0.23
	1	0.26
	1	0.26
mean	1.00	0.25
s.e.	0.00	0.02

**Table S12 (Fig. S1d) Decreases in ADC values of staurosporine-treated HeLa cells**		
	ADC ($\times 10^{-3}$ mm^2/s)	
	control	24 h
	0.80	0.62
	0.79	0.62
	0.80	0.61
mean	0.80	0.62
s.e.	0.00	0.01

Table S13 (Fig. 4b) Early increases (8 h) and subsequent decreases (48 h) in cell volume of TNFα-treated L929 cells					
	ADC (x 10^{-3} mm^2/s)				
	0 h	3 h	8 h	24 h	48 h
	1.57	1.47	2.18	1.64	1.16
	1.50	1.56	2.12	1.54	1.22
	1.56	1.55	2.15	1.53	1.15
	1.27	1.43		1.65	1.15
mean	1.48	1.50	2.15	1.59	1.17
s.e.	0.14	0.06	0.03	0.06	0.03

Table S14 (Fig. 4c) Early increases (3 h and 8 h) and subsequent decreases (24 h and 48 h) in cell viability of TNFα-treated L929 cells					
	cell viability (vs. 0 h)				
	0 h	3 h	8 h	24 h	48 h
	1	1.11	1.21	0.55	0.16
	1	1.15	1.26	0.59	0.17
	1	1.20	1.45	0.61	0.17
mean	1.00	1.15	1.31	0.59	0.17
s.e.	0.00	0.04	0.13	0.03	0.01

Table S15 (Fig. 4f) Gradual increase in ADC value of necroptotic L929 cells					
	ADC (x 10^{-3} mm^2/s)				
	0 h	3 h	8 h	24 h	48 h
	0.78	0.76	0.88	0.90	0.99
	0.79	0.80	0.88	0.92	1.08
	0.80	0.80	0.89	0.94	1.09
mean	0.79	0.79	0.88	0.92	1.05
s.e.	0.01	0.02	0.01	0.02	0.06

Table S16 (Fig. S2b) Decreases in cell volume of z-VAD-fmk-treated (20 μM) L929 cells		
	cell volume (pL)	
	control	48 h
	1.77	0.88
	1.83	1.00
	1.84	1.08
mean	1.81	0.99
s.e.	0.04	0.10

Table S17 (Fig. S2c) Decreases in cell viability of z-VAD-fmk-treated (20 μM) L929 cells		
	cell viability (vs. control)	
	control	48 h
	1	0.09
	1	0.10
	1	0.11
mean	1.00	0.10
s.e.	0.00	0.01

Table S18 (Fig. S2d) Increases in ADC values of z-VAD-fmk-treated (20 μM) L929 cells		
	ADC ($\times 10^{-3}$ mm^2/s)	
	control	48 h
	0.83	0.99
	0.80	1.02
	0.89	1.02
mean	0.84	1.01
s.e.	0.05	0.02

Table S19 (Fig. S3b) Dose-dependent increases in cell volume (= cell swelling) of irradiated (5–40 Gy) U937 cells

	cell volume (pL)									
	0 Gy	5 Gy			20 Gy			40 Gy		
	0 h	72 h	120 h	144 h	72 h	120 h	144 h	72 h	120 h	144 h
	1.98	2.98	1.91	2.23	4.96	2.78	1.99	4.63	4.17	3.01
	2.06	2.97	1.90	2.09	4.82	2.61	2.61	4.66	3.66	2.68
	2.09	2.88	2.23	2.31	4.77	2.25	2.04	4.77	3.66	2.87
mean	2.04	2.94	2.01	2.21	4.85	2.55	2.21	4.69	3.83	2.85
s.e.	0.06	0.06	0.19	0.11	0.10	0.27	0.34	0.07	0.29	0.17

Table S20 (Fig. S3c) Time-dependent decreases in cell viability of L929 cells treated with x-irradiation (20 Gy)

	cell viability at 20 Gy (vs. 0 Gy)					
	72 h		96 h		120 h	
	0 Gy	20 Gy	0 Gy	20 Gy	0 Gy	20 Gy
	1	0.52	1	0.28	1	0.17
	1	0.57	1	0.30	1	0.17
	1	0.57	1	0.29	1	0.17
mean	1.00	0.57	1.00	0.29	1.00	0.17
s.e.	0.00	0.02	0.00	0.01	0.00	0.00

Table S21 (Fig. S3f)										
	ADC (×10^{-3} mm^2/s)									
	control	5 Gy			20 Gy			40 Gy		
	0 h	72 h	120 h	144 h	72 h	120 h	144 h	72 h	120 h	144 h
	0.69	0.60	0.68	0.74	0.65	0.76	0.84	0.69	0.75	0.87
	0.69	0.60	0.68	0.73	0.67	0.74	0.83	0.71	0.73	0.86
	0.69	0.60	0.69	0.75	0.67	0.75	0.85	0.71	0.74	0.87
mean	0.69	0.60	0.68	0.74	0.66	0.75	0.84	0.70	0.74	0.87
s.e.	0.00	0.00	0.00	0.01	0.01	0.01	0.01	0.01	0.01	0.00

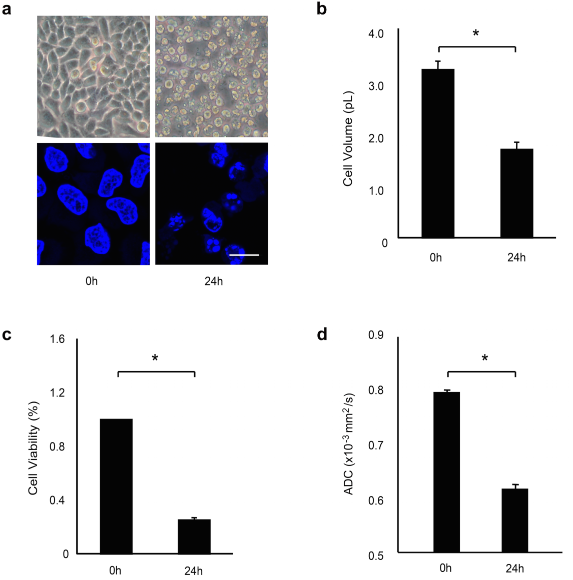

a
0h
24h
b
Cell Volume (pL)
4.0
3.0
2.0
1.0
0
*
0h
24h
c
Cell Viability (%)
1.6
1.2
0.8
0.4
0
*
0h
24h
d
ADC (x10-3 mm2/s)
0.9
0.8
0.7
0.6
0.5
*
0h
24h

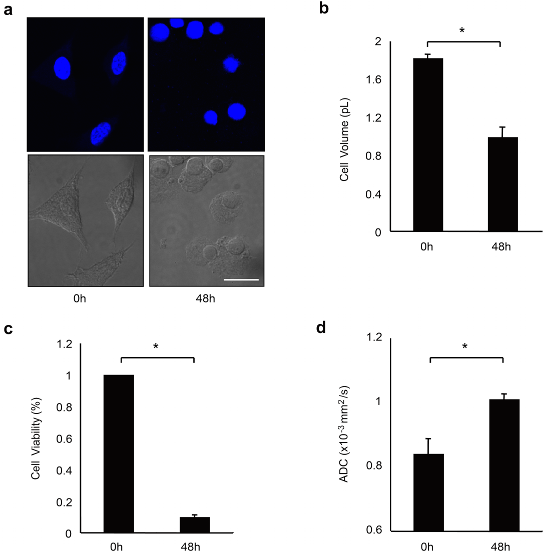
a
0h
48h
b
*
Cell Volume (pL)
2
1.6
1.2
0.8
0.4
0
0h
48h
c
*
Cell Viability (%)
1.2
1
0.8
0.6
0.4
0.2
0
0h
48h
d
*
ADC (x10-3 mm2/s)
1.2
1
0.8
0.6
0h
48h

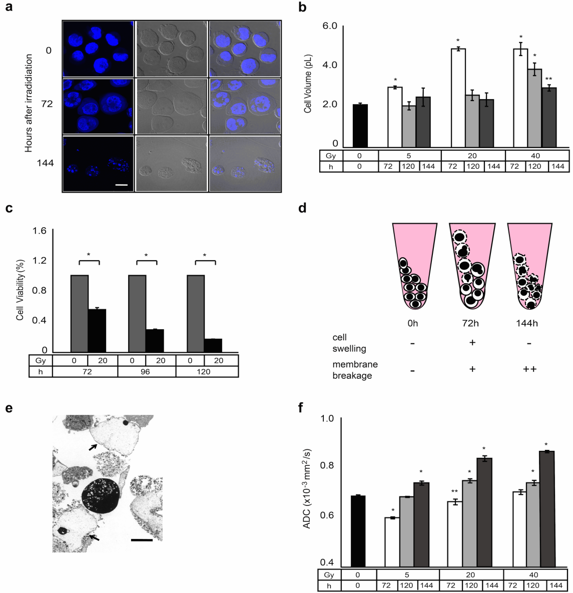
a
Hours after irradidiation
0
72
144
b
Cell Volume (pL)
6.0
4.0
2.0
0
Gy 0 5 20 40
h 0 72 120 144 72 120 144 72 120 144
c
Cell Viability (%)
1.6
1.2
0.8
0.4
0
Gy 0 20 0 20 0 20
h 72 96 120
d
0h
72h
144h
cell swelling - + -
membrane breakage - + ++
e
f
ADC (x10^{-3} mm^2/s)
1.0
0.8
0.6
0.4
Gy 0 5 20 40
h 0 72 120 144 72 120 144 72 120 144

Figure legends for supplementary figures

Fig. S1. Molecular diffusion of staurosporine-treated (apoptotic) HeLa cells

a, Phase contrast microscopy (upper panels) and confocal microscopy for DAPI staining (lower panels) showing fragmented nuclei of staurosporine-treated (1 μM) HeLa cells. Scale bar = 20 μm

b, Graph showing decreases in the cell volume of staurosporine-treated HeLa cells. * Significantly different (p <0.001; t-test).

c, Graph showing decreases in the cell viability of staurosporine-treated HeLa cells. * Significantly different (p <0.001; t-test).

d, Graph showing decreases in the ADC values of staurosporine-treated HeLa cells. * Significantly different (p <0.001; t-test).

Fig. S2. Molecular diffusion of z-VAD-fmk-treated (necroptotic) L929 cells

a, Confocal microscopy for DAPI staining and differential interference microscopy showing intact nuclei of z-VAD-fmk-treated (20 μM) L929 cells. Scale bar = 20 μm.

b, Graph showing decreases in the cell volume of z-VAD-fmk-treated (20 μM) L929 cells. * Significantly different (p <0.001; t-test).

c, Graph showing decreases in the cell viability of z-VAD-fmk-treated (20 μM) L929 cells. * Significantly different (p <0.001; t-test).

d, Graph showing increases in the ADC values of z-VAD-fmk-treated (20 μM) L929 cells. * Significantly different (p <0.001; t-test).

Fig. S3.—Molecular diffusion of irradiated U937 cells

a, Confocal microscopy for DAPI staining showing chromatin clustering of irradiated (20 Gy) U937 cells (144 h). Scale bars = 20 μm.

b, Graph showing dose-dependent increases in the cell volume (= cell swelling) of

irradiated (5–40 Gy) U937 cells. *, ** Significantly different from cell volume at 0 h (* p <0.001, **p <0.01; Tukey-Kramer test).

c, Graph showing time-dependent decreases in the cell viability of L929 cells treated with x-irradiation (20 Gy). * Significantly different (p <0.001; t-test).

d, Schematic representation and cellular characteristics of cell pellets containing irradiated (72 h and 144 h after 20-Gy irradiation) in culture medium.

e, Transmission electron microscopy showing plasma membrane disruption with extensive outflows of nuclei and intracellular organelles (arrows). Scale bar = 5 μm.

f, Graph showing early (72 h) decreases and subsequent (120 h, 144 h) increases in the ADC values of irradiated (5–40 Gy) U937 cells. *, ** Significantly different from the ADC values at 0 h (* p <0.001, ** p <0.05; Tukey-Kramer test).
